# Additional hepatic ^166^Ho-radioembolization in patients with neuroendocrine tumours treated with ^177^Lu-DOTATATE; a single center, interventional, non-randomized, non-comparative, open label, phase II study (HEPAR PLUS trial)

**DOI:** 10.1186/s12876-018-0817-8

**Published:** 2018-06-15

**Authors:** Arthur J. A. T. Braat, Dik J. Kwekkeboom, Boen L. R. Kam, Jaap J. M. Teunissen, Wouter W. de Herder, Koen M. A. Dreijerink, Rob van Rooij, Gerard C. Krijger, Hugo W. A. M. de Jong, Maurice A. A. J. van den Bosch, Marnix G. E. H. Lam

**Affiliations:** 1 Department of Radiology and Nuclear Medicine, University Medical Centre Utrecht, Heidelberglaan 100, 3584 CX Utrecht, the Netherlands; 2 Department of Nuclear Medicine, Erasmus Medical Centre, ‘s-Gravendijkwal 230, 3015 CE Rotterdam, the Netherlands; 3 Department of Endocrinology, Erasmus Medical Centre, ‘s-Gravendijkwal 230, 3015 CE Rotterdam, the Netherlands; 4 Department of Endocrinology, VU University Medical Centre Amsterdam, De Boelelaan 117, 1081 HV Amsterdam, the Netherlands

**Keywords:** NET, Neuroendocrine tumour, Liver metastasis, Radioembolization, Holmium-166, PRRT, Lutetium-177, ^177^Lu-DOTATATE

## Abstract

**Background:**

Neuroendocrine tumours (NET) consist of a heterogeneous group of neoplasms with various organs of origin. At diagnosis 21% of the patients with a Grade 1 NET and 30% with a Grade 2 NET have distant metastases. Treatment with peptide receptor radionuclide therapy (PRRT) shows a high objective response rate and long median survival after treatment. However, complete remission is almost never achieved. The liver is the most commonly affected organ in metastatic disease and is the most incriminating factor for patient survival. Additional treatment of liver disease after PRRT may improve outcome in NET patients. Radioembolization is an established therapy for liver metastasis. To investigate this hypothesis, a phase 2 study was initiated to assess effectiveness and toxicity of holmium-166 radioembolization (^166^Ho-RE) after PRRT with lutetium-177 (^177^Lu)-DOTATATE.

**Methods:**

The HEPAR PLUS trial (“***H****olmium* ***E****mbolization* ***P****articles for* ***A****rterial* ***R****adiotherapy* ***P****lus* ^*177*^***Lu****-DOTATATE in* ***S****alvage NET patients”*) is a single centre, interventional, non-randomized, non-comparative, open label study. In this phase 2 study 30–48 patients with > 3 measurable liver metastases according to RECIST 1.1 will receive additional ^166^Ho-RE within 20 weeks after the 4th and last cycle of PRRT with 7.4 GBq ^177^Lu-DOTATATE. Primary objectives are to assess tumour response, complete and partial response according to RECIST 1.1, and toxicity, based on CTCAE v4.03, 3 months after ^166^Ho-RE. Secondary endpoints include biochemical response, quality of life, biodistribution and dosimetry.

**Discussion:**

This is the first prospective study to combine PRRT with ^177^Lu-DOTATATE and additional ^166^Ho-RE in metastatic NET. A radiation boost on intrahepatic disease using ^166^Ho-RE may lead to an improved response rate without significant additional side-effects.

**Trial registration:**

Clinicaltrials.gov NCT02067988, 13 February 2014. Protocol version: 6, 30 november 2016.

## Background

In accordance with the most recent WHO/ENETS criteria, grade 1 and 2 neuroendocrine tumours (G1-/G2NET) are regarded as well- to moderately-differentiated tumours and grade 3 NET (G3NET) as poorly-differentiated NET or neuroendocrine carcinomas (NEC) [1, 2]. At diagnosis, 21% of all G1NET, 30% of all G2NET and 50% of all G3NET have distant metastases, of which the liver is most commonly affected [3, 4]. A correlation between the organ of origin and the likelihood of metastasis exists. For example, rectal NET has a slim chance of distant metastasis (5%) compared with pancreatic or colonic NET (respectively 64% and 53–86%) [3, 5]. Considering these numbers, many patients will be ineligible for curative treatment, which currently only includes surgical resection of the primary tumour.

**Fig. 1:**
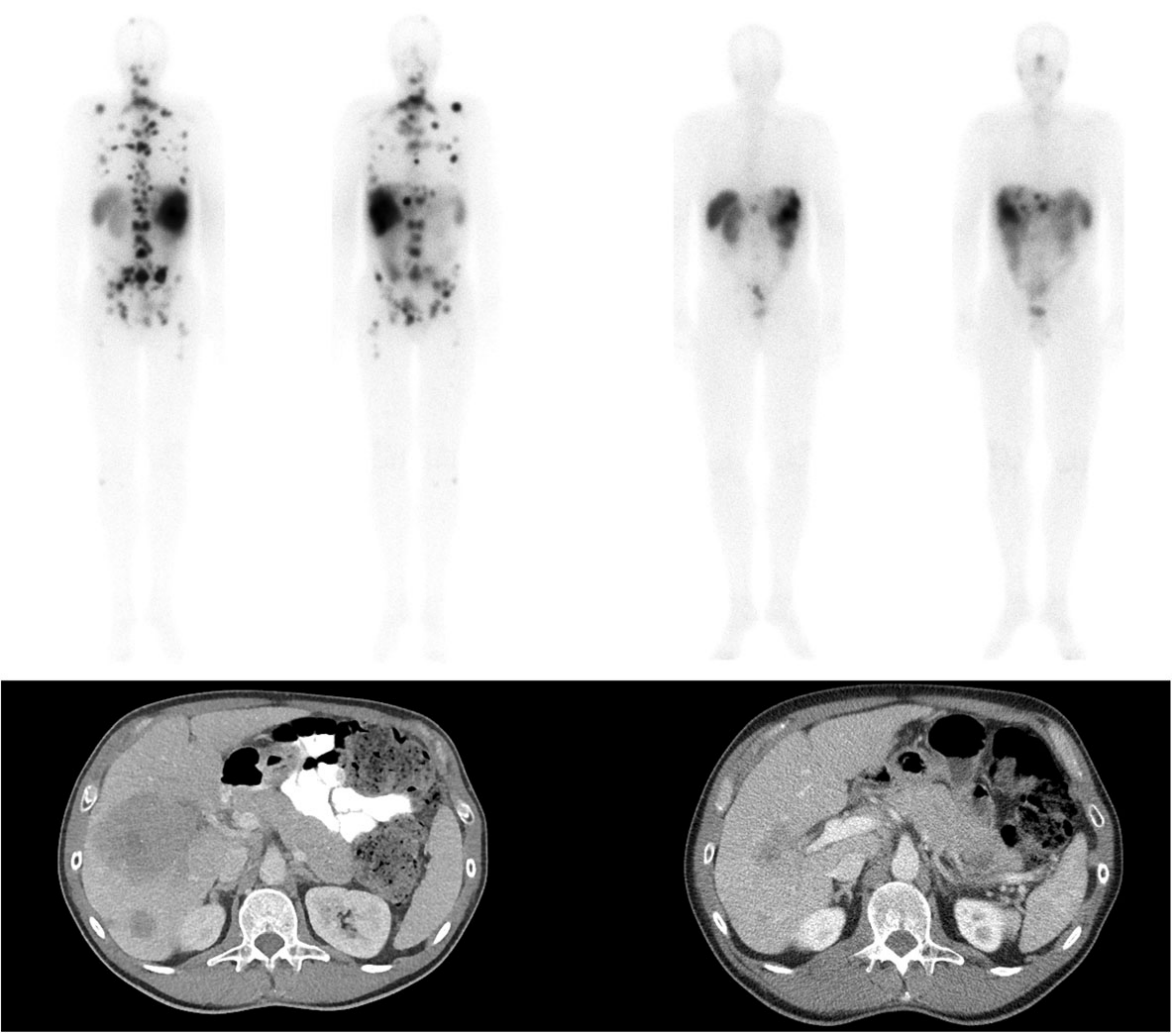
Example of ^177^Lu-DOTATATE in NET. Upper row: planar whole body ^111^In-pentetreotide scintigraphy. Lower row: venous phased CT of the liver. On the left baseline imaging and on the right imaging after ^177^Lu-DOTATATE treatment

Most G1-/G2NET have membrane receptors for somatostatin, allowing for targeted therapies, of which somatostatin-analogs are the most commonly used (e.g. octreotide). Treatment with somatostatin-analogs, chemotherapeutics and kinase inhibitors show only limited objective response rates in G1-/G2NET [6–12]. In addition, systemic therapies give rise to systemic side effects. In the last decade, the treatment of G1-/G2NET with peptide receptor radionuclide therapies (PRRT) has increased. High objective imaging response rates (CR + PR 29–58%) [13–17], clinical and biological response rates and a long median survival (95–128 months after diagnosis, 46 months after treatment) [13, 14] can be achieved after PRRT (Fig. 1).

**Fig. 2:**
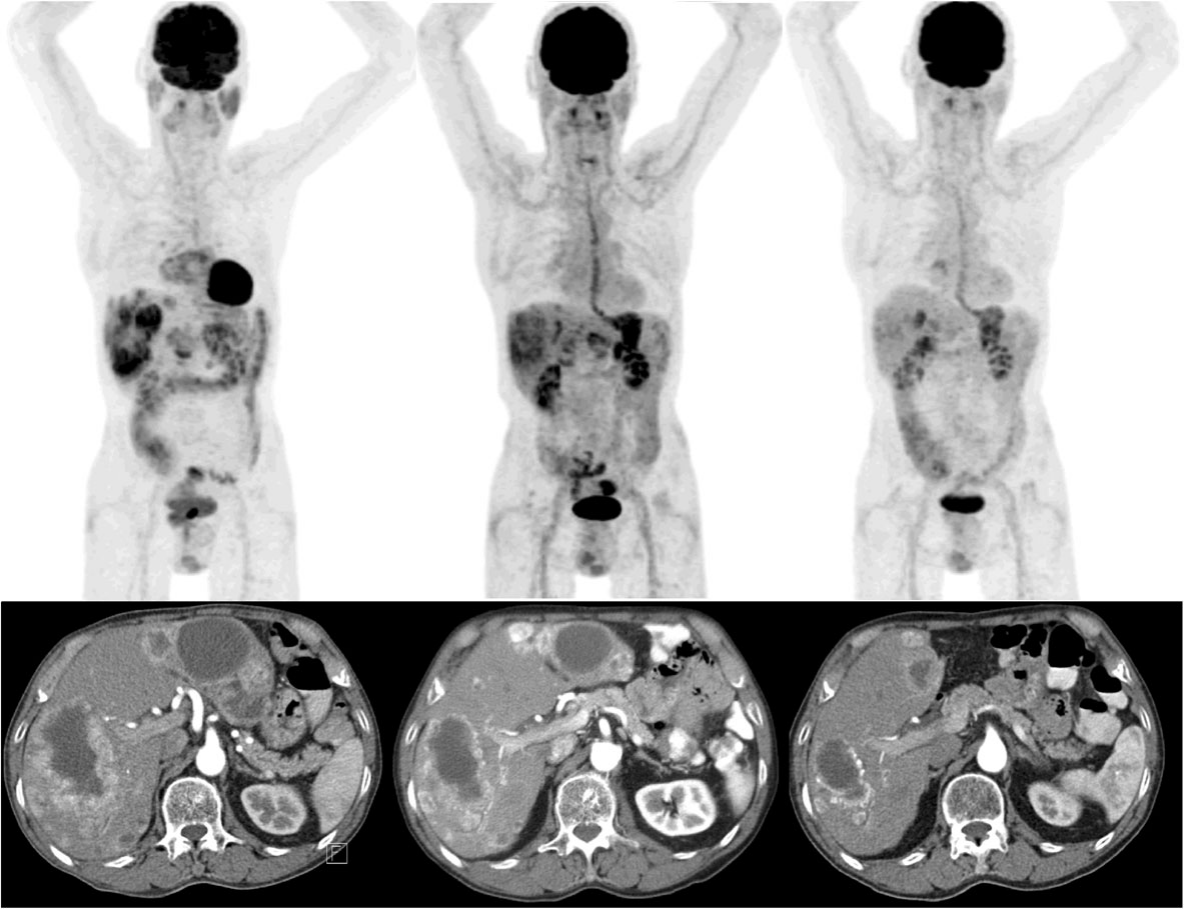
Example of ^166^Ho-radioembolization in NET. A patient with a grade 2 small intestinal NET according to the WHO-criteria, treated in the prior HEPAR 2 trial. On the left, the ^18^FDG-PET and venous phased CT at baseline. In the middle, the imaging studies 3 months after ^166^Ho-RE with partial metabolic ^18^FDG-PET response and some tumour reduction on CT. On the right, follow-up imaging studies 6 months after ^166^Ho-RE with significant partial metabolic response and significant tumour reduction on CT

All studies include high percentages of patients with liver metastases and show a dismal prognosis with increasing liver involvement (Table 1). As surgical resection techniques develop, some forms of hepatic involvement can be treated surgically. However, as three different patterns of hepatic metastases are described in NET, patients with the most common ‘diffuse pattern’ are not eligible for surgical resection (Table 2) [4]. Besides, most systemic therapies have a limited objective response rate. This indicates a need for improved treatment of extensive liver disease. In accordance with the ENETS guideline published in 2012, the treatment of choice in patients with NET liver metastases with a ‘diffuse’ or unresectable ‘complex pattern’, consists of systemic treatment followed by liver directed treatment [4]. Hepatic radioembolization (RE) is one of the liver directed treatments and it is an established minimal invasive treatment of patients with liver malignancies. RE has been demonstrated to be effective and well tolerated in primary, as well as secondary liver malignancies. A recent meta-analysis by Devcic et al. showed an average objective response rate (CR + PR) of 50% and an average disease control rate of 86% in a heterogeneous group of NET treated with RE [18]. ^166^Ho-RE is quite similar to ^90^Y-RE, but its distinct advantages will be discussed later on. Figure 2 shows an example of ^166^Ho-RE in a NET patient.

**Table 1:** Liver involvement as a poor prognostic factor in different therapeutic studies

Author	Treatment	N	Liver involvement	Median survival (months)	5-year survival	*p* values
Chamberlain (2000) [46]	Surgical resection	85	0–25%	–	90%	
			25–50%	–	83%	
			50–75%	47	80%	
			> 75%	24	–	
Yao (2001) [47]	Surgical resection	16	≤4 liver metastases	46	–	< 0.05
			> 4 liver metastases	20	–	
Gupta (2005) [48]	TAE or TACE	123	0–25%	86	–	
			25–50%	30	–	< 0.10
			50–75%	39	–	< 0.17
			> 75%	20	–	< 0.05
Kwekkeboom (2008) [14]	PRRT	310	None	> 48	–	
			Moderate	> 48	–	
			Extensive	25	–	< 0.01

Legend: *TAE* transarterial (bland) embolization, *TACE* transarterial chemoembolization, *PRRT* peptide receptor radionuclide therapy

**Table 2:** NET Liver involvement patterns [4]

	Involvement	Incidence
Simple pattern	One lobe or two adjacent lobes	20–25%
Complex pattern	Primarily one lobe and smaller satellites contralaterally	10–15%
Diffuse pattern	Multifocal disease	60–70%

In the current clinical setting, RE is used for liver dominant or liver isolated disease, often in a salvage setting. In this study, it is hypothesized that improved outcome can be obtained by escalating the treatment of liver metastases, the most significant prognostic factor for NET patients, additional to treatment of all extrahepatic disease in G1-/G2NET patients, by combining systemic PRRT with RE. In the presented study, patients with metastasized NET will receive PRRT in 4 cycles of 7.4 GBq with ^177^Lu-DOTATATE, followed by ^166^Ho-RE in the University Medical Centre Utrecht, the Netherlands, using ^166^Ho-microspheres. The following paragraphs will address the details of the study.

## Methods

### Study design

The HEPAR PLUS study is a single centre, interventional, non-randomized, non-comparative, open label study. In this phase 2 study all patients will receive additional ^166^Ho-RE after ^177^Lu-DOTATATE. Overall, 30–48 patients with metastasized NET will be investigated for efficacy and toxicity.

### Subjects

Patients with NET and liver metastasis, who completed 4 cycles of 7.4 GBq ^177^Lu-DOTATATE, will receive additional ^166^Ho-RE within 20 weeks of the last/fourth cycle of ^177^Lu-DOTATATE. At time of recruitment, all included patients have no need for conventional treatment options like surgery or chemotherapy. In the Netherlands, ^177^Lu-DOTATATE is often a first- or second-line treatment. Previous treatments prior to ^177^Lu-DOTATATE were no exclusion criterium. Detailed inclusion and exclusion criteria are listed in Table 3.

**Table 3:** Inclusion and exclusion criteria

Inclusion criteria	Exclusion criteria
Patient must have given a written informed consent	Brain metastases or spinal cord compression, unless irradiated > 4 weeks prior to ^166^Ho-RE and stable for at least 1 week without steroids
≥ 18 years of age	Serum bilirubin > 1.5 x upper limit of normal
Confirmed histological diagnosis NET	Glomerular filtration rate < 35 ml/min
Prior treatment with 4 cycles of 7.4 GBq ^177^Lu-DOTATATE within 20 weeks before ^166^Ho-RE	Alkaline phosphatase, alanine aminotransferase or aspartate aminotransferase > 5 x upper limit of normal
Life expectancy > 12 weeks	Leucocytes < 2.0 × 10^9^/l and/or platelet count < 50 × 10^9^/l
WHO performance score 0–2	Significant cardiac event within 3 months of inclusion
≥ 3 measurable liver lesions according to RECIST 1.1	Patients suffering from diseases with an increased chance of liver toxicity
Negative pregnancy test for women of childbearing potential	Patients declared incompetent or suffering from psychic disorders making comprehensive judgment impossible
No nursing activities for women of childbearing potential	Severe bile duct abnormalities: papillotomy, cholecystectomy, biliary stents and bilidigestive anastomosis are allowed
Acceptable method of contraception	Body weight > 150 kg
	Severe contrast allergy
	Liver tumour involvement > 70% on CT

### Time schedule

Recruitment will take place between Augustus 2014 and January 2019. First participant was enrolled in November 2014.

### Medical device

^166^Ho-microspheres are produced by incorporating non-radioactive ^165^Ho and its acetylacetonate complex (^165^HoAcAc) in a poly(L-lactic acid) matrix to form microspheres with an average diameter of 30 μm. By neutron-activation in a nuclear facility, the prescribed amount of radioactive ^166^Ho-microspheres are produced [19, 20]. The radionuclide ^166^Ho has a half-life of 26.8 h, is a beta-emitter (Ε_βmax_ = 1.85 MeV) and a gamma emitter (Ε_γ_ = 81 keV). Due to their additional photon emitting properties, ^166^Ho-microspheres can be visualized and quantified using SPECT imaging [21, 22].

### Recruitment

All patients have previously been treated with four cycles of ^177^Lu-DOTATATE. After the fourth cycle patients are eligible for study inclusion. The study physician (AJATB) and principal investigator (MGEHL) inform all patients; thereafter informed consent will be obtained. On the informed consent form, participants can indicate whether they wish to receive a summary of the trial results, once the trial is completed.

### Statistical analysis

This single arm open label study will have a sequential design. Stopping boundaries are determined such that an overall one-side alpha of at the most 0.05 is maintained in case the true tumour response is 20%. Early termination at a response interim analysis (after 30, 36 or 42 patients) is determined by pre-defined boundaries on the number of partial and complete responses according to RECIST 1.1 (Table 4). A superiority or futility boundary may be reached or crossed before 30 patients are reached, but the study will continue to at least 30 patients to allow estimation of the key secondary endpoints. The sequential design with boundaries as given in Table 4 will have a power of 90% to reach a positive tumour response decision in case the true target lesions tumour response is 40%. The exact overall one-sided type I error is 4.5%.

**Table 4:** Stopping boundaries for early termination at interim analysis

Analysis	Sample Size	Lower boundary	Upper boundary
1	30	5	11
2	36	6	13
3	42	7	14
4	48	15	16

Interim analysis of toxicity with descriptive statistics (N, mean, median, etc.) will be performed for every 3 patients. All analysis will be performed in the Full Analysis Set (FAS), including all patients who received at least the scout dose procedure (see below). The Per Protocol Set (PPS) will include all patients who complied with the protocol up to at least 3 months. PPS analyses will be used for the primary endpoint. For the assessment of the primary objective at least 30 patients should have a 3 months follow-up CT-scan. If patients do not reach the 3 months follow-up CT-scan or receive a new treatment prior to the evaluation moment, a new patient will be included for the PPS analysis.

### Monitoring

All safety interim analyses will be presented to our Independent Data Monitoring Commission (IDMC), consisting of one interventional radiologist, one nuclear medicine physician, one gastroenterologist and one biostatistician. All IDMC members are not involved in the trial and have no conflicting interests. Additionally, safety analysis will be performed every 3 months during the recruitment of the first 30 patients, after patient 36, patient 42 and patient 48, and evaluated by the IDMC.

Severe adverse (device) events will be reported to the Ethics Committee of the University Medical Center Utrecht and IDMC within 8 days. In accordance with Dutch regulations on research with medical devices, a summary of all severe adverse (device) events will be reported to the Dutch Health Care Inspectorate (in Dutch: Inspectie GezondheidsZorg en Jeugd in oprichting; IGJ) every 3 months.

### Data management

All patient data collected in this trial, will be coded. All coded data will be entered in to an, in-house developed, electronic case report form (e-CRF) in a secure digital environment. All collected data is monitored and validated by an independent, external data monitor approximately every 3–4 months. In accordance with Dutch regulations, all collected data will be stored for a duration of 15 years. Trial data is only accessible to the study physician, principal investigator and external data monitor.

### Ethical considerations

The study protocol has been approved by the Ethics Committee and the institutional radiation protection committee of the University Medical Centre Utrecht, the Netherland. This study will be performed in accordance with the Declaration of Helsinki (current version October 2013), the Medical Research Involving Human Patients Act (WMO, the Netherlands) and the requirements of International Conference on Harmonization (Good Clinical Practice). In accordance to regulations, all future protocol amendments need Ethics Committee approval. Unexpected harm to the participant during the trial, is covered by the institutions’ insurance for clinical trials.

### Funding

This phase 2 study is funded by the Department of Radiology and Nuclear Medicine of the University Medical Center Utrecht. No external funding received.

### Treatment

#### Screening

After obtaining informed consent, all study proceedings will occur in the University Medical Center Utrecht, Utrecht, The Netherlands. A screening visit will take place at the outpatient clinic prior to the first angiography. The study physician and principal investigator will check in- and exclusion criteria, perform a physical examination (including blood pressure, temperature and heart rate) and assess the WHO performance status of the patient. All patients are asked to fill out the European Organization for Research and Treatment of Cancer (EORTC) QLQ-C30 and QLQ-GINET21 questionnaires. Additional tests include relevant laboratory testing (haematology, coagulation profile and serum chemistry) including a tumour marker (when present/measurable in the patient), electrocardiogram (ECG) and a contrast-enhanced CT. All contrast enhanced CT’s will be assessed by RECIST 1.1 criteria [23].

#### Angiography

Patients are admitted for 3 days (2 nights) starting the day prior to the angiography. After physical examination and relevant laboratory testing, patients are pre-hydrated to prevent kidney damage, and started on proton pump inhibitors for 6 weeks (pantoprazole once a day 40 mg). Premedication 1 h prior to the angiography consists of one dose of corticosteroids, antihistamines and anti-emetics (respectively dexamethasone 10 mg, clemastine 1 mg and ondansetron 8 mg), at the same time a tranquilizer is offered to the patient (oxazepam 10 mg). If the patient is familiar with a mild to moderate contrast allergy, additional corticosteroids and antihistamines will be given prior to the angiography according with national guidelines of the Central Accompaniment Institution (CBO in Dutch) [24].

A skilled and trained interventional radiologist will perform all angiographies of the upper abdominal vessels. A catheter is introduced via one of the femoral arteries by the Seldinger technique. After identifying all arteries supplying the liver, additional branches of these arteries that supply other organs than the liver are coiled, if needed. This usually involves the gastroduodenal artery (GDA) and right gastric artery (RGA).

#### Scout dose

Once successful identification of the supplying arteries and occlusion of additional branches has been performed, a scout dose of 250 MBq ^166^Ho-microspheres will be administered [20, 25]. Due to the photon emission of ^166^Ho, distribution of the microspheres, lung shunting and extrahepatic depositions can all be assessed using SPECT/CT. Planar imaging and SPECT/CT will be performed following the angiography and evaluated qualitatively as well as quantitatively. Extrahepatic deposition of activity is a contra-indication for treatment. Lung shunting will be assessed by planar imaging and SPECT/CT and should not exceed the maximum tolerable lung absorbed dose (i.e. 30 Gy).

#### Treatment

**Fig. 3:**
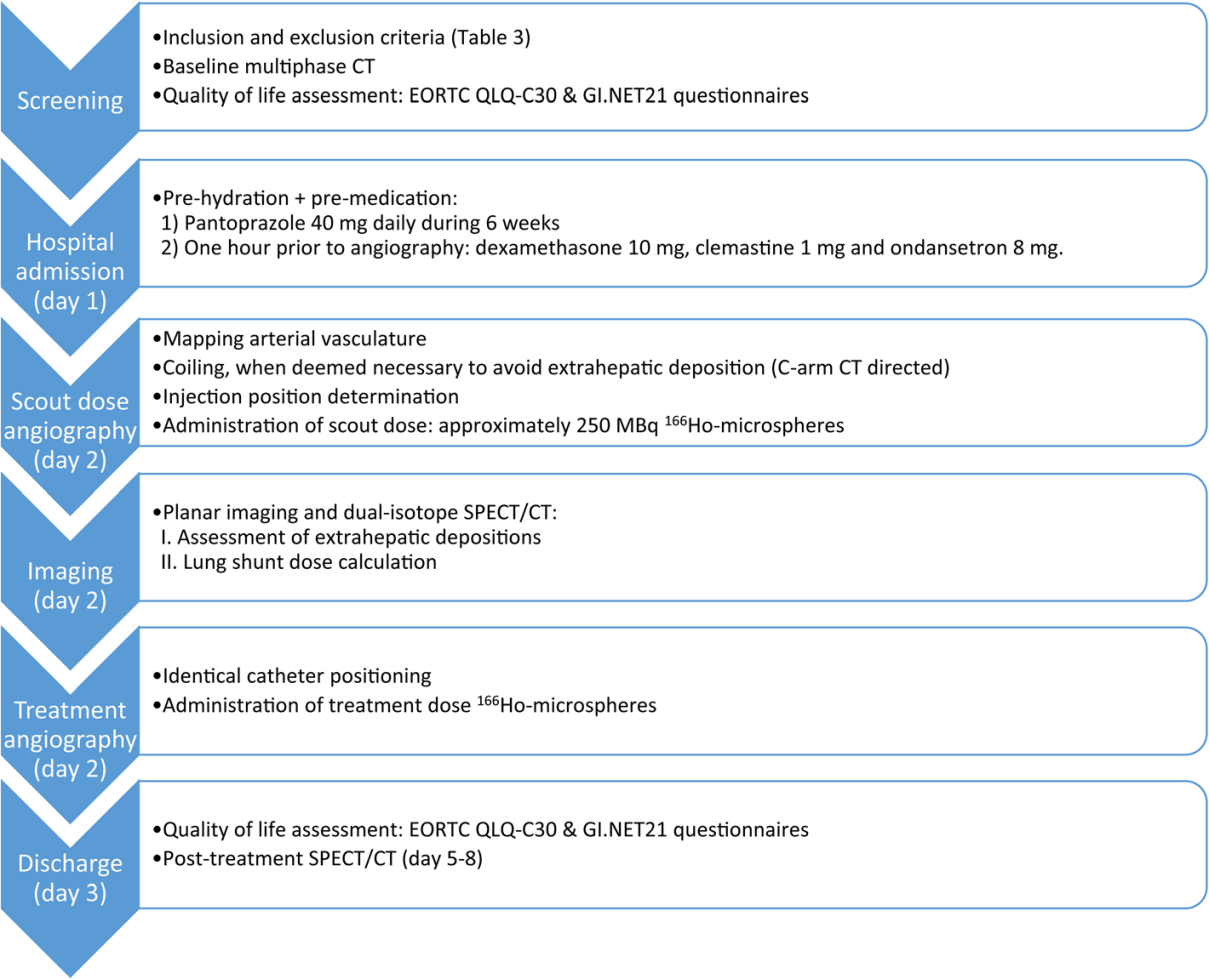
Study protocol depicting the time line and study proceedings between inclusion and hospital discharge

If the pre-treatment assessment is successful, patients return to the angiography suite for the treatment angiography combined with the ^166^Ho-RE. This will take place on the same day as the pre-treatment angiography and scout dose procedure (see Fig. 3). Based on the results of the dose escalation study (i.e. HEPAR I trial), a whole liver absorbed dose of 60 Gy was determined to be safe. A whole liver absorbed dose of 60 Gy leads to the following equation for activity calculation:

$$ {A}_{{}^{166} Ho}(MBq)=3781\ \left( MBq\ast \frac{Gy}{J}\right)\ast liver\ weight\ (kg) $$

As mentioned above, additional information derived from the scout dose SPECT/CT can change treatment planning, either performing a one session whole liver treatment or two session sequential whole liver treatment. A significant lung shunt dose (> 30 Gy) will lead to a reduction in treatment activity.

#### Radiation exposure rate

The radiation exposure rate of the patient will be measured from 1-m distance at *t* = 0 h and *t* = 24 h after the ^166^Ho-RE.

#### Follow-up

##### Follow-up visits

During 12 months after treatment, patients are followed at the outpatient clinic. The visits will take place after 3 weeks, 6 weeks, 3 months, 6 months, 9 months and 12 months (closing visit). During these visits patients will undergo a physical examination, laboratory testing, WHO performance status assessment and will be monitored for (serious) adverse (device) events. Prior to the 3 weeks, 6 weeks and 3 months visits, patients are asked to fill out the EORTC questionnaires (QLQ-C30 and QLQ-GI.NET21). Prior to the 3, 6, 9 and 12 months visits, a CT will be performed for response assessment according to the RECIST 1.1 criteria.

##### Primary objectives

Two distinct objectives are the focus of our study. Tumour response on CT at 3 months of follow-up will be the first primary objective. This is defined as complete response (CR = disappearance of all lesions) or partial response (PR = ≥30% decrease in the sum of the longest diameters of the target lesions, compared to baseline measurements). The second primary objective is to establish the safety and toxicity profile of treatment with ^166^Ho-RE as an additional treatment after ^177^Lu-DOTATATE, using the Common Terminology Criteria for Adverse Events (CTCAE version 4.03) [26].

##### Secondary objectives

Three secondary objectives have been defined. Anti-tumour effect will be assessed by relevant tumour markers (when available), expressed as a percentage of the pre-treatment values. Furthermore, Quality of Life (QoL) will be assessed using the EORTC questionnaires (QLQ-C30 and QLQ-GI.NET21) during the first 3 months after treatment. The impact of treatment on QoL will be compared to tumour response and other parameters.

Additionally, biodistribution and dosimetry will be evaluated using a dual isotope fusion SPECT/CT protocol. After the standard scout dose SPECT/CT and treatment dose SPECT/CT (i.e. ^166^Ho-SPECT), 50 MBq of ^99m^Tc-phytacis (CIS bio, France) will be administered. Subsequently a dual-isotope SPECT/CT will be acquired, simultaneously providing a ^166^Ho-SPECT for assessment of microsphere distribution and a ^99m^Tc-phytacis SPECT for the assessment of truly functional liver parenchyma.

##### Safety profile

The phase 1 study on ^166^Ho-RE (HEPAR I trial) [20] and its subsequent phase 2 study (HEPAR 2 trial) [27], demonstrated similar treatment-related effects as the current commercially available ^90^Y-microspheres. Common adverse events up to grade 1 or 2 of the CTCAE v4.03 included: fever, nausea, vomiting, abdominal discomfort, and fatigue, often called the post-embolization syndrome. These complaints were generally self-limiting within 4–6 weeks. More serious adverse events of RE in general were rare (< 1%) and included RE-induced liver disease (REILD) [28] and inadvertent extrahepatic distribution of activity [29].

##### Escape medication

The protocol ensures all patients are pre- and post-hydrated in order to minimize the chance of renal insufficiency caused by the vascular contrast agent, jodixanol (Visipaque®). After ^166^Ho-RE standard escape medication includes paracetamol up to 4000 mg / day and ondansetron up to 24 mg, as respectively oral analgesic and intravenous anti-emetic. If persisting nausea occurs, additional metoclopramide up to 120 mg / day will be used. In case of diarrhoea, patients will receive loperamide up to 16 mg / day. In this specific patient group, some patients might experience excessive release of NET-related hormones that could cause a ‘carcinoid syndrome’ or ‘carcinoid crisis’. These complaints can be prevented (to some extent) with octreotide intravenously, steroids and hydration.

##### Withdrawal of individual patients

Patients may be withdrawn from the study if a serious adverse event occurs.

Patients will be withdrawn from the study if 1) the investigator considers it in the best interest of the patient that he/she be withdrawn (e.g. progressive disease), 2) the patient withdraws consent or 3) the patient is unable to comply with the protocol procedures.

## Discussion

Liver metastases significantly limit patient survival (Table 1). In the current study, the beneficial effect of additional ^166^Ho-RE within 12 weeks after systemic ^177^Lu-DOTATATE will be investigated. Combining these treatments may lead to an improved response rate for liver metastases, with acceptable and suppressible side effects, which may eventually lead to prolonged survival. Although the latter question is not an objective of the current phase 2 study, if significant efficacy and limited toxicity are shown, a subsequent phase 3 study might be initiated.

To date, Ezziddin et al. published the only report describing RE with ^90^Y-microspheres after PRRT with ^177^Lu-DOTATATE in a retrospective study [30]. They described a population of 23 patients in which RE was performed in a salvage setting. Patients had progressive or functionally uncontrolled disease after PRRT. Three months after RE, 30% had PR and 61% had stable disease without any serious toxicity, comparable with other reports on ^90^Y-microspheres in NET patients. The authors concluded that salvage RE after PRRT shows a toxicity profile similar to RE alone, despite the high cumulative activity administrated. Less than 15% experienced a CTCAE grade 3 toxicity (abdominal pain, fatigue, fever, nausea and vomiting) and one patient developed a gastroduodenal ulcer. The interval between PRRT and RE was not mentioned, but patients were only referred for RE in case they had progressive disease (i.e. salvage setting). In their study, a cumulative liver dose of 2–12 Gy was described after PRRT. Due to the hypervascular nature of NET, the absorbed dose on healthy liver parenchyma due to RE is (far) below the presumed toxicity limit of healthy liver tissue (i.e. 70 Gy; and 50 Gy in cirrhotic livers with ^90^Y resin microspheres) [31]. Thus, in theory, combining RE and PRRT can be safe. Nonetheless, concerns arise when implementing RE shortly after PRRT, due to the cumulative radiation dose and the short interval, potentially provoking REILD [28]. On the other hand in a recent case report, Filippi et al. described their treatment combination a patient with one hepatic metastasis, mesenteric metastasis and several bone metastases, diagnosed on a ^68^Ga-DOTATATE-PET/CT [32]. The hepatic metastasis was downstaged with a lobar RE procedure, followed by 4 cycles of PRRT to treat extrahepatic lesions (a mesenteric metastasis and several bone metastases). Restaging after 3 months with a ^68^Ga-DOTATATE-PET/CT showed a nearly complete remission of the extrahepatic metastases and an incomplete remission of the hepatic metastasis, thus another additional lobar RE procedure was performed to treat the hepatic lesion, with success [32]. They reported no significant adverse events of the combined treatments, complete symptomatic control and a survival of 42 months [32]. Additionally, they reported an absorbed dose on healthy liver parenchyma after the RE procedures of just 18 Gy and 20 Gy [32]. An example that the combination of RE and PRRT with short intervals can be safe.

In contrast to other studies, ^99m^Tc-macroaggregated albumin (^99m^Tc-MAA) will not be used as a scout dose for treatment planning. Instead, a small number of ^166^Ho-microspheres will be used as a scout dose (approximately 250 MBq). This may overcome known limitations of ^99m^Tc-MAA: 1) differences in flow dynamics caused by the randomly shaped ^99m^Tc-MAA particles (90% between 10 and 90 μm) versus the spherically shaped ^166^Ho-microspheres (30 ± 5 μm), 2) differences in scanning protocols of pre- and post-procedural imaging (i.e. ^99m^Tc-SPECT vs ^90^Y-PET versus ^166^Ho SPECT for both procedures), 3) employing a similar injection technique during both angiographies (i.e. bolus ^99m^Tc-MAA versus intermittent injection of ^166^Ho microspheres), and 4) overestimation of the lung shunt using ^99m^Tc-MAA.

**Fig. 4:**
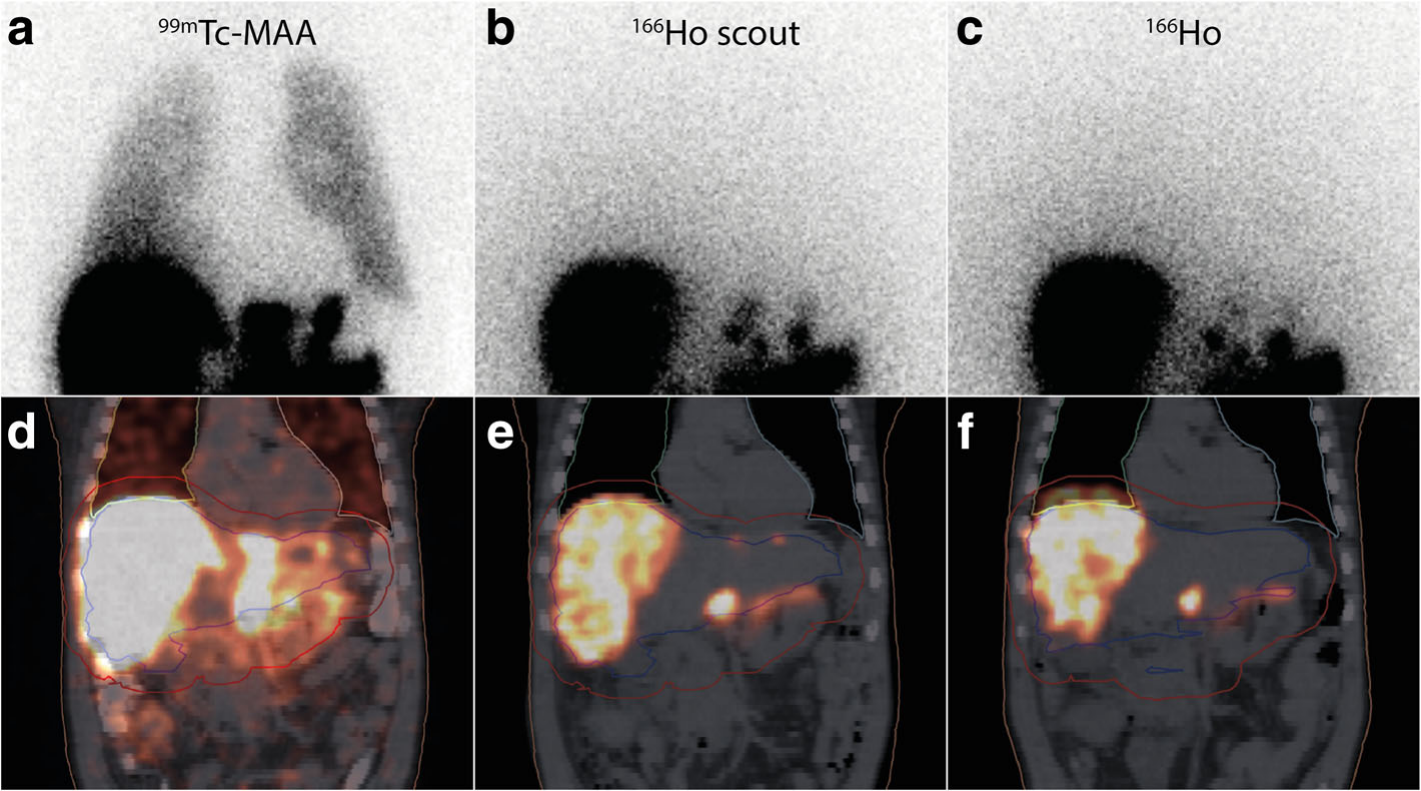
Example of lung shunt fraction overestimation by ^99m^Tc-MAA. A patient with multiple liver metastases of a cholangiocarcinoma treated in the prior HEPAR 2 trial. Note the (visual) significant overestimation of ^99m^Tc-MAA on planar imaging compared to the ^166^Ho-scout dose and ^166^Ho-treatment dose. Quantification of the lung shunt fraction on planar and SPECT/CT imaging confirmed the visual assessment: **a** ^99m^Tc-planar = 13.4%, **d** ^99m^Tc-SPECT = 6%, all ^166^Ho imaging modalities (**b**, **c**, **e** and **f**) with the scout dose and with the treatment dose showed a lung shunt fraction of < 1%

As shown by Elschot et al., in patients treated with ^166^Ho-RE, using ^99m^Tc-MAA as well as ^166^Ho-microspheres as pre-treatment imaging scout dose, ^166^Ho-SPECT/CT was the most accurate in predicting the lung absorbed dose after ^166^Ho-RE [33]. On ^166^Ho-SPECT/CT a median lung shunt dose of 0.02 Gy was calculated. This was significantly overestimated in lung shunt dose calculations based on ^99m^Tc-MAA planar scintigraphy (5.5 Gy), ^166^Ho planar scintigraphy (10.4 Gy) and ^99m^Tc-MAA SPECT/CT (2.5 Gy). An example of severe overestimation by ^99m^Tc-MAA compared to ^166^Ho-microsperes is shown in Fig. 4. In the present study biodistribution / extrahepatic deposition assessment and lung shunt dose calculation will solely be evaluated by ^166^Ho-SPECT/CT.

The safety of administrating 250 MBq of beta-emitting ^166^Ho-microspheres as a scout dose has been studied by Prince et al. [25] They predicted the amount of extrahepatic activity and radiation absorbed dose, using ^99m^Tc-MAA SPECT/CT of 160 patients prior to ^90^Y-RE. Based on a prior study by Kao et al., they defined a dose exceeding 49 Gy as clinically significant [25, 34, 35]. Simulating the use of 250 MBq ^166^Ho-microspheres as a scout dose, only 1.3% of the patients had an extrahepatic deposition that could potentially be harmful (i.e. exceeded a mean dose of 49 Gy) [25]. Additionally, C-arm CT’s will be acquired at each injection position, prior to scout dose administration, to minimize the chance of extrahepatic depositions and partial tumour coverage, and to avoid extra angiography procedures [36, 37].

As mentioned in the ‘secondary objectives’ section, the application of the dual isotope SPECT/CT protocol will enable us to derive all relevant dosimetric parameters for treatment dose calculation. ^99m^Tc-phytacis, like other radiocolloids, is extracted from the blood pool by the reticuloendothelial cells of the liver [38]. Solely the functional liver parenchyma is depicted, due to absence of reticuloendothelial cells in tumours. Validation of this dual-isotope protocol will be performed in a side-study. Previous studies by Lam et al. have shown the prognostic value of the combination of ^99m^Tc-MAA and ^99m^Tc-sulphur colloid imaging, showing a tumour dose-response correlation and healthy liver dose-toxicity correlation [39, 40].

As an additional advantage, holmium is one of the 14 lanthanide elements, making ^166^Ho-microspheres MRI-compatible for treatment imaging. In short, using estimated R_2_* value changes from multi-gradient echo data, the holmium concentration per voxel could be determined [41, 42]. Conversion into units of activity enables dosimetric calculations. A prior study by Smits et al. has shown its feasibility in clinical practice and its comparability to SPECT-based dosimetry [21]. Using a similar MR-sequence, real-time imaging of the ^166^Ho-microspheres during administration may become a future application [43]. However, the use of MRI is beyond the scope of this study, to minimize the study impact for patients.

In contrast to RE, PRRT’s main limitation is absorbed kidney dose. To reduce the dose, the most important preparatory measure is intravenous amino acid infusion prior, during and after ^177^Lu-DOTATATE administration. To overcome the disadvantage of the unavoidable kidney dose, intra-arterial administration of ^177^Lu-DOTATATE in patients with liver only disease could be a future application [44]. Several studies show a decreased absorbed kidney dose and an increased uptake in the liver metastases in most patients after intra-arterial administration. In a study by Pool et al., kidney absorbed dose decreased by 13% in addition to a 2.9-fold increase in liver metastases uptake [45]. In theory, a combination of intra-arterial PRRT and RE could be superior in patients with liver only disease.

Limitations of the study protocol are the small study cohort, non-comparative design, single center design and relatively short clinical follow-up for NET patients.

In conclusion, combining PRRT and RE could lead to improved treatment response and additional survival benefit: PRRT can be used to treat intra- and extrahepatic disease, whereas RE leads to an additional radiation boost on intrahepatic disease, the most incriminating factor in NET-patients’ survival. Based on this hypothesis, the HEPAR PLUS trial will include all patients treated with ^177^Lu-DOTATATE with significant intrahepatic disease.

### Recruitment status

Ongoing: Currently recruiting patients.

## Abbreviations

^166^Ho: ^166^Holmium isotope
^177^Lu: ^177^Lutetium isotope
^90^Y: ^90^Yttrium isotope
^99m^Tc-MAA: ^99m^Technetium-macroalbumin aggregates
CR: complete response
CTCAE: Common Terminology Criteria for Adverse Events
ECG: Electrocardiogram
ENETS: European Neuroendocrine Tumour Society
EORTC: European Organisation for Research and Treatment of Cancer
GBq: Gigabecquerel
Gy: Gray
MBq: Megabecquerel
NET: Neuroendocrine tumour
PR: Partial response
PRRT: Peptide receptor radionuclide therapy
QoL: Quality of Life
RE: Radioembolization
RECIST: Response Evaluation Criteria In Solid Tumours
SPECT/CT: Single photon emission computed tomography + computed tomography
WHO: World Health Organization
